# Heterocellular N-cadherin junctions enable nontransformed cells to inhibit the growth of adjacent transformed cells

**DOI:** 10.1186/s12964-021-00817-9

**Published:** 2022-02-17

**Authors:** Stephanie A. Sheehan, Edward P. Retzbach, Yongquan Shen, Harini Krishnan, Gary S. Goldberg

**Affiliations:** 1 Department of Molecular Biology and Graduate School of Biomedical Sciences, Rowan University School of Osteopathic Medicine, Stratford, NJ 08084 USA; 2 Department of Physiology and Biophysics, Stony Brook University, Stony Brook, NY 11794 USA

**Keywords:** Cadherins, Cancer, Cell junctions, Contact normalization, Oral squamous cell carcinoma, Podoplanin

## Abstract

**Background:**

The Src tyrosine kinase phosphorylates effector proteins to induce expression of the podoplanin (PDPN) receptor in order to promote tumor progression. However, nontransformed cells can normalize the growth and morphology of neighboring transformed cells. Transformed cells must escape this process, called “contact normalization”, to become invasive and malignant. Contact normalization requires junctional communication between transformed and nontransformed cells. However, specific junctions that mediate this process have not been defined. This study aimed to identify junctional proteins required for contact normalization.

**Methods:**

Src transformed cells and oral squamous cell carcinoma cells were cultured with nontransformed cells. Formation of heterocellular adherens junctions between transformed and nontransformed cells was visualized by fluorescent microscopy. CRISPR technology was used to produce cadherin deficient and cadherin competent nontransformed cells to determine the requirement for adherens junctions during contact normalization. Contact normalization of transformed cells cultured with cadherin deficient or cadherin competent nontransformed cells was analyzed by growth assays, immunofluorescence, western blotting, and RNA-seq. In addition, Src transformed cells expressing PDPN under a constitutively active exogenous promoter were used to examine the ability of PDPN to override contact normalization.

**Results:**

We found that N-cadherin (N-Cdh) appeared to mediate contact normalization. Cadherin competent cells that expressed N-Cdh inhibited the growth of neighboring transformed cells in culture, while cadherin deficient cells failed to inhibit the growth of these cells. Results from RNA-seq analysis indicate that about 10% of the transcripts affected by contact normalization relied on cadherin mediated communication, and this set of genes includes PDPN. In contrast, cadherin deficient cells failed to inhibit PDPN expression or normalize the growth of adjacent transformed cells. These data indicate that nontransformed cells formed heterocellular cadherin junctions to inhibit PDPN expression in adjacent transformed cells. Moreover, we found that PDPN enabled transformed cells to override the effects of contact normalization in the face of continued N-Cdh expression. Cadherin competent cells failed to normalize the growth of transformed cells expressing PDPN under a constitutively active exogenous promoter.

**Conclusions:**

Nontransformed cells form cadherin junctions with adjacent transformed cells to decrease PDPN expression in order to inhibit tumor cell proliferation.

**Plain English Summary:**

Cancer begins when a single cell acquires changes that enables them to form tumors. During these beginning stages of cancer development, normal cells surround and directly contact the cancer cell to prevent tumor formation and inhibit cancer progression. This process is called contact normalization. Cancer cells must break free from contact normalization to progress into a malignant cancer. Contact normalization is a widespread and powerful process; however, not much is known about the mechanisms involved in this process. This work identifies proteins required to form contacts between normal cells and cancer cells, and explores pathways by which cancer cells override contact normalization to progress into malignant cancers.

**Graphical abstract:**

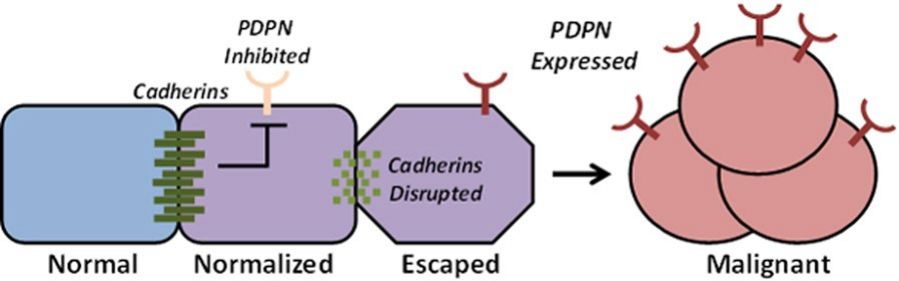

**Video Abstract**

**Supplementary Information:**

The online version contains supplementary material available at 10.1186/s12964-021-00817-9.

## Background

Elucidating effects of oncogenic protein kinase activity on cell growth is a relatively direct task in cultured cell lines. Results from these studies indicate that Src and other tyrosine kinases phosphorylate effectors including the Crk associated substrate/breast cancer antiestrogen resistance protein 1 (p130Cas/BCAR1) adaptor protein to induce expression of the podoplanin (PDPN) receptor in order to promote transformed cell growth and expansion [1–5]. However, interactions between neighboring cells in the tumor microenvironment complicate this scenario.

Nontransformed cells can normalize the growth and morphology of neighboring transformed cells. This process, called “contact normalization”, can control the growth of cells transformed by chemical carcinogens and oncogenic kinases including Src [6–9]. Contact normalization controls cell behavior downstream of Src kinase activity to inhibit expression of effectors including PDPN [4, 5, 10–12].

Communication by diffusible factors is not sufficient to mediate contact normalization. Direct heterocellular junctions between transformed and nontransformed cells are required for this process. However, specific junctions that mediate contact normalization have not been identified [6–8]. Connexins [13, 14] and cadherins [15–17] have been implicated in growth control by intercellular communication.

Connexins form gap junctions that enable neighboring cells to share intracellular signals including metabolites and ions in a controlled fashion. In general, gap junctional communication is disrupted between cancer cells compared to their nontransformed precursors, and connexins have been identified as tumor suppressors [13, 14]. There are functional relationships between mitogenic kinase activity and gap junctional communication. For example, Src phosphorylates Cx43 and p130Cas/BCAR1, which causes them to associate with Crk and inhibit junctional communication [18–20]. However, Cx43 can form channels between Src transformed and nontransformed cells, and this heterocellular communication correlates with contact normalization [21, 22]. Nonetheless, while connexin activity correlates with contact normalization, it is not required. Results from studies with Cx43 knockout cells exposed to a chemical channel blocker indicate that nontransformed cells can normalize the growth and morphology of adjacent Src transformed cells in the absence of gap junctional communication [12].

Cadherins form intercellular adherens junctions that utilize catenins to link the actin cytoskeletons of adjacent cells to establish and maintain normal multicellular tissue architecture. E-cadherin (E-Cdh) and N-cadherin (N-Cdh) are commonly expressed “classical” cadherins that can act as tumor suppressors [15–17, 23, 24]. As with connexins, the Src kinase can phosphorylate cadherins and associate proteins (catenins) to disrupt adherens junctions and promote tumor progression [25–27]. Here, we report that heterocellular cadherin junctions are required for contact normalization, and that forced PDPN expression can override this form of growth suppression.

## Methods

### Cell lines and maintenance

Cells were cultured in DMEM (Mediatech, inc., 10–014-CV) supplemented with 25 mm HEPES (Mediatech, inc., 25-060-Cl), and 10% FBS (Serum Source International, Inc., DH5293) at 37 °C, 5% CO_2_, and 100% humidity. Nontransformed mouse embryonic cells (MECs), Src transformed MECs (MEC-Src) [4, 12], and human oral squamous cell carcinoma cells (HSC-2) [28] have been previously described. Nontransformed N-Cdh knockout (MEC-Cdh2Ko) cells were produced from MECs using N-Cdh CRISPR/Cas9 (Santa Cruz #sc-419593) and N-Cdh HDR plasmids (Santa Cruz #sc-419593-HDR) with Plasmid Transfection Medium (Santa Cruz #sc-108062) and UltraCruz Transfection Reagent (Santa Cruz #sc-395739). Transfected cells were selected in puromycin, visually confirmed by fluorescent microscopy, and N-Cdh deletion was confirmed by western blotting with N-Cdh antibody (BD Biosciences #610920). Homozygous null PDPN knockout mouse embryonic fibroblasts were transfected with v-Src kinase in pBABEpuro and PDPN in pEF4Zeo [29–31] with lipofectamine 2000 (Invitrogen #52758) and selected in Zeocin and puromycin to produce Src transformed MECs with constitutive PDPN expression (MEC-SrcPdpn cells).

Fluorescently tagged cadherins were generated by transfection with mammalian expression vectors as fusion proteins. The coding sequences for N-Cdh-YFP and N-Cdh-CFP fusion proteins were released from pEcfpCdh2YFP and pEcfpCdh2CFP [32] (provided by Kathleen Green, Northwestern University) with Bgl2/Not1 and inserted into the BamH1/Not1 site of pEF4/V5-His A (Invitrogen V941-20) to produce pEf4Cdh2YFP and pEf4Cdh2CFP, respectively. These expression constructs were transfected into nontransformed (Cdh2YPF) and Src transformed (Cdh2CFP) MECs with lipofectamine 2000 (Thermofisher #11668) and selected by zeocin resistance (Invivogen, ant-zn-1p).

### Live and immunofluorescent cell imaging

Cells were cultured on 35 mm poly-d-lysine-coated glass bottom culture dishes (MatTek Corp., P35GC-1.5-14-C) and visualized directly, or stained with Hoechst (Molecular Probes 33342), fixed in paraformaldehyde, blocked with BSA, and incubated with antibody specific for N-cdh (Santa Cruz Biotechnology D-4 #Sc-8424), mouse PDPN (University of Iowa Developmental Studies Hybridoma Bank #8.1.1), human PDPN (Bio-Rad D2-40 #MCA2543), and p120-catenin (Cell Signaling Technology #59854). Primary antibodies were detected with appropriate secondary antibodies including Alexa Fluor 488 goat anti-mouse (Invitrogen #A11001), Alexa Fluor 647 goat anti-mouse (Invitrogen #A-21235), Alexa Fluor 488 goat anti-rabbit (Invitrogen #A-11034), and Alexa Fluor 647 goat anti-hamster (Invitrogen #A-21451). For some experiments, transformed cells were stained with DiI (ThermoFischer Scientific #D282) before plating to identify them in cocultures with nontransformed cells at a 1:300 ratio as described [4, 12]. Cells were visualized on a Carl Zeiss Axio Observer Z1 equipped with a Plan-Apochromat 63X objective, apotome 2, filter sets to detect CFP (excitation 436 ± 20, emission 480 ± 40), Alexa Fluor 488, and YFP (excitation 470 ± 40, emission 525 ± 50), DiI (excitation 560 ± 40, emission 630 ± 75), Hoechst (excitation 390 ± 22, emission 460 ± 50), and Alexa Fluor 647 (excitation 640 ± 30, emission 690 ± 50) with a Zeiss AxioCam Mrc camera Rev3 equipped with an INU series Tokai Hit Stage Top Incubator and Zen Pro 2.3 software as previously described [4, 28, 29].

### Cell growth assays and layered culture system

To examine the effect of nontransformed cells on the growth of neighboring transformed cells, transformed cells were labeled with DiI and plated at a 1:300 ratio with nontransformed cells. Nontransformed cells plated alone, and DiI labeled transformed cells plated 1:300 with respective nonlabelled transformed cells, were used as controls. Cultures were grown for 7 days and medium was replenished every 2 days to minimize depletion of serum growth factors. The number of transformed cells in a 0.242 mm^2^ microscopic field were counted on day 7 using a Zeiss Axiovert 40 CFL equipped with a Plan-Apochromat 20X objective and filter sets to detect DiI fluorescence (excitation, 560 ± 540; emission, 630 ± 675) with a Zeiss AxioCam Mrc camera equipped with Axiovision software as previously described [4, 28, 29].

A layered culture system was used to allow separated populations of transformed and nontransformed cells to form intercellular junctions with each other as described [4, 10, 12]. Briefly, 100,000 Src transformed (MEC-Src), human OSCC (HSC-2) cells, or nontransformed (MEC) cells were plated on porous membranes (Costar 3542) containing 300,000 nontransformed (MEC or MEC-CdhKo) or transformed (MEC-Src) cells on the other side. Transformed and nontransformed cells form intercellular junctions with each other through the 3 micron pore size in the membrane which prevent cells from actually migrating to the other side. Cells were harvested and analyzed 24 h after plating.

### Western blotting

Western blotting was performed as previously described [28, 29]. Briefly, protein from cells lysed in lysis buffer (2% SDS, 1% glycerol, 50 mM DTT in 62.5 mM Tris–HCl, pH 6.8) was resolved by 10% SDS-PAGE (18ug/lane), transferred to Immobilon-P membranes (EMD Millipore #IPVH00010), and incubated with antisera specific for mouse PDPN (University of Iowa Developmental Studies Hybridoma Bank #8.1.1), human PDPN (BioRad #D2-40 and NZ-1 from Yukinari Kato), p120-catenin (Cell Signaling Technology #59854), GAPDH (Santa Cruz #FL335), β-actin (Sigma #A1978), Pan-Cdh (Sigma #C1821), E-Cdh (BD Biosciences #610181), N-Cdh (BD Biosciences #610920), v-Src (Sigma Millipore #05-185/EC10), and active Src kinase (phosphorylated at Tyr 416, Cell Signaling Technology #2101). Primary antiserum was recognized by appropriate secondary antiserum specific for mouse (Invitrogen #31430), rabbit (Santa Cruz #sc-2305), rat (Sigma Millipore #AP136P), or Syrian hamster (Santa Cruz #sc-2493) IgG conjugated to horseradish peroxidase and detected by enhanced chemiluminescence (Thermo Scientific #32209). Membranes were stained with India ink and protein gels were stained with Coomassie blue to verify equal loading and transfer. Western blot signals from control and experimental groups were quantitated from the same blot by image densitometry (ImageJ) and normalized to control values for comparisons between experiments.

### RNA-Seq analysis

Nontransformed (MEC) and Src transformed (MEC-Src) cells cultured with themselves, and Src transformed (MEC-Src) cells were cultured with cadherin competent (MEC) and deficient (MEC-CdhKo) nontransformed nontransformed cells in the layered culture system. RNA was extracted from samples using Qiagen RNeasy Plus Universal mini kit according to the manufacturer’s instructions (Qiagen, Hilden, Germany), and RNA sequencing libraries were prepared using the NEBNext Ultra RNA Library Prep Kit for Illumina using manufacturer’s instructions (NEB, Ipswich, MA, USA) by GENEWIZ, LLC. (South Plainfeld, NJ, USA) as previously described [33]. Sequencing libraries were clustered on a single lane of a flowcell and loaded on an Illumina HiSeq instrument according to manufacturer’s instructions. The samples were sequenced using a 2 × 150 bp Paired End (PE) configuration. Image analysis and base calling were conducted by HiSeq Control Software (HCS). Raw sequence data (.bcl files) generated from Illumina HiSeq were converted into fastq files and de-multiplexed using Illumina's bcl2fastq 2.17 software. One mismatch was allowed for index sequence identification. Raw data were converted to transcripts per million (TPM) values, and transcripts were filtered based on an initial TPM of equal to or greater than 1 in any sample group. Data from this study can be accessed at Sequence Read Archive (SRA) submission: SUB10367693. Protein interaction networks were identified using STRING (https://string-db.org/) with a minimum required interaction score of 0.400 (medium), 0.700 (high), and 0.900 (highest). Gene Ontology (GO) enrichment analyses was performed using the gene ontology resource (http://geneontology.org/) and BioMart Ensembl (https://m.ensembl.org/biomart/).

### Statistical analysis

Two-tailed Student’s t-test was used to identify differences between values. Number of repeats and *p* values are presented in figure legends describing each experiment. Excel (Microsoft) and Prism (Graphpad) software was used for analyses.

### Ethics

The study involved only murine and established human cells without patient identifiers.

## Results

**Fig. 1:**
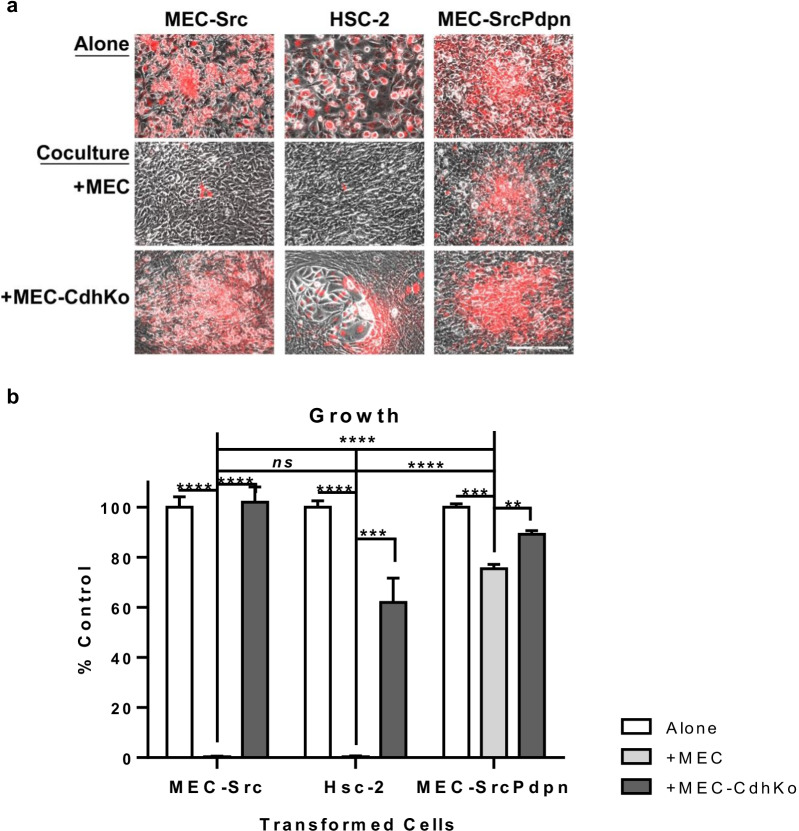
Cadherins are required for contact normalization. **a** Src transformed mouse embryonic cells (MEC-Src), human OSCCs (HSC-2), and Src transformed cells transfected with PDPN (MEC-SrcPdpn) were labeled with DiI (red). These labeled transformed cells were then plated at a 1:300 ratio with unlabeled transformed cells, nontransformed wild type mouse embryonic cells (MEC), or cadherin knockout mouse embryonic cells (MEC-CdhKo). Cultures were grown for 7 days and visualized by phase contrast and fluorescence microscopy as indicated (scale bar = 500 µm). **b** The number of transformed (red) cells in a 425 × 570 µm field were counted and shown as percent of transformed cells plated alone (mean + SEM, n = 4). Double, triple, and quadruple asterisks indicate differences with *p* < 0.01, *p* < 0.001, and *p* < 0.0001, respectively, by t-test as indicated

Intercellular junctions are needed to mediate contact normalization. Although these junctions have not yet been identified, gap junctions formed by connexins have been ruled out by process of elimination [8, 12, 34]. We utilized well characterized gap junction deficient nontransformed and Src transformed MECs (mouse embryonic cells) [4, 8, 12] to investigate the role of intercellular junctions in contact normalization. Nontransformed MECs inhibited the growth of Src transformed MECs in coculture by over 90% in this model system as shown in Fig. 1.

### Adherens junctions are required for contact normalization

**Fig. 2:**
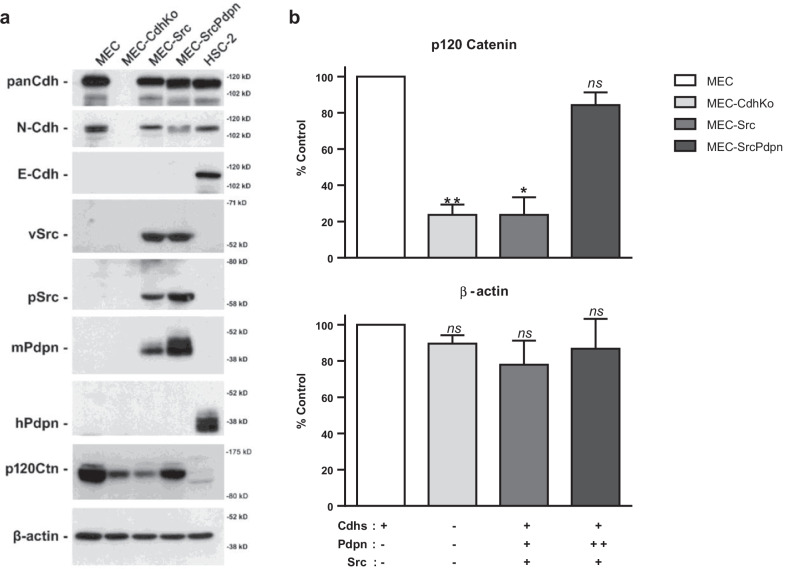
Cadherin, PDPN, p120-catenin, Src kinase, and β-actin expression in transformed and nontransformed cells. **a** Pan-Cdh, N-Cdh, E-Cdh, v-Src, active Src (phosphorylated at Tyr 416), mouse and human PDPN, p120-catnin (p120Ctn), and β-actin were detected by Western blot analysis of protein from nontransformed (MEC and MEC-CdhKo) and transformed (MEC-Src, MEC-SrcPdpn, and HSC-2) cells as indicated. **b** Western blot results were quantitated and shown as percent of nontransformed MEC controls (mean + SEM, n = 3). Single asterisk, double asterisks, and ns indicate differences with *p* < 0.05, *p* < 0.01, or *p* > 0.05, respectively, by t-test as indicated

Cadherins form adherens junctions which have profound effects on cell adhesion, morphology, motility, and proliferation. Many tumor cells and neighboring stromal cells express N-Cdh. Transformed cells are initially surrounded by nontransformed cells in their microenvironment. Tumor cells can influence cadherin expression in the microenvironment as cancer progresses. For example, tumor cells can convert neighboring fibroblasts into cancer associated fibroblasts (CAFs). N-Cdh expression in tumor cells and CAFs is often associated with increased cell motility and metastasis [8, 17, 35, 36]. However, the role of N-Cdh junctions between tumor cells and nontransformed cells has not been thoroughly defined. We examined the effects of N-Cdh expression in well characterized transformed and nontransformed MECs in this study [4, 5, 10, 12, 33]. These cells expressed N-Cdh as detected by Western blotting shown in Fig. 2a.

**Fig. 3:**
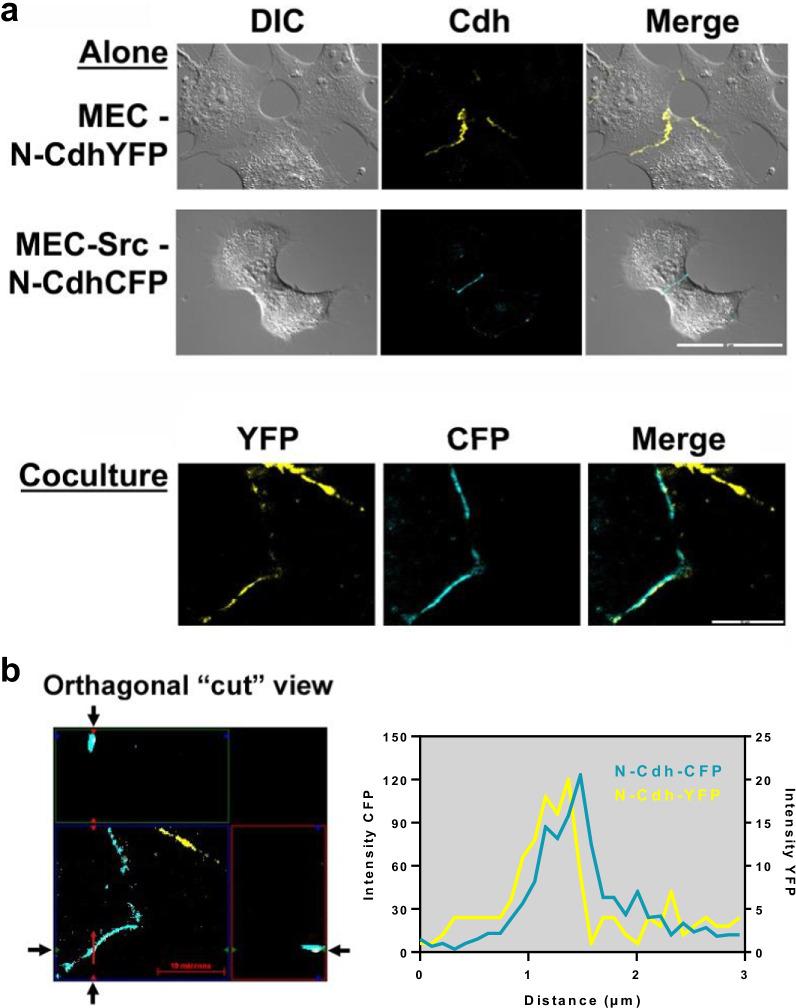
Heterocellular N-cadherin junctions form between nontransformed and transformed cells. **a** Live cell imaging of nontransformed MECs transfected with N-Cdh-YFP and MEC-Src cells transfected with N-Cdh-CFP in monoculture (scale bar = 50 μm) or coculture (scale bar = 20 μm) was performed by confocal microscopy with DIC, fluorescent, and merged images shown as indicated. Colocalized heterocellular N-Cdh junctions appear white in cocultured cells. **b** Orthogonal imaging of N-Cdh-YFP and N-Cdh-CFP colocalization in cut out view (scale bar = 10 μm) with intensity plot profile (arbitrary units) over distance in one focal plane of an observed area as indicated

Having found N-Cdh expressed by both transformed and nontransformed MECs, we sought to determine if these cadherins formed heterocellular junctions. Src transformed and nontransformed cells were transfected with YFP and CFP labeled N-Cdh, respectively, and analyzed by live cell microscopy. Imaging of cells expressing these fluorescently tagged fusion constructs revealed that N-Cdh in transformed and nontransformed cells colocalized with each other at areas of intercellular contact as shown in Fig. 3. These results indicate that heterocellular adherens junctions formed between nontransformed and Src transformed cells.

After identifying N-Cdh junctions formed between transformed and nontransformed cells, we sought to determine if they were required for contact normalization. We utilized CRISPR to delete N-Cdh expression in nontransformed MECs to produce MEC-CdhKo (cadherin knockout) cells. These cells were cadherin deficient as confirmed by Western blot analysis with cadherin antibodies shown in Fig. 2a. Cadherin ablation rendered these nontransformed cells unable to inhibit the growth of Src transform cells in coculture as shown in Fig. 1. These data indicate that nontransformed cells formed adherens junctions with adjacent transform cells to inhibit their growth by contact normalization.

Having identified N-Cdh as the intercellular junction responsible for contact normalization of Src transformed MECs, we sought to investigate the generality of this scenario. We utilized HSC-2 human OSCC (oral squamous cell carcinoma) cells for this study since they express both N-Cdh and E-Cdh as shown in Fig. 2a. Similar to Src transformed MECs, nontransformed MECs inhibited the growth of these human OSCC cells in coculture by over 90% as shown in Fig. 1.

Following confirmation that nontransformed MECs inhibited the growth of HSC-2 cells, we investigated the role of N-Cdh in this model of transspecies contact normalization. While wild type MECs decreased cocultured HSC-2 cell growth by over 90%, cadherin deficient MEC-CdhKo cells decreased neighboring HSC-2 cell growth by about 40%. This difference was significant, with *p* < 0.001 (n = 4) as shown in Fig. 1. These data indicate that N-Cdh mediated contact normalization of both Src transformed and human OSCC cells.

### Podoplanin expression is sufficient to override contact normalization

**Fig. 4:**
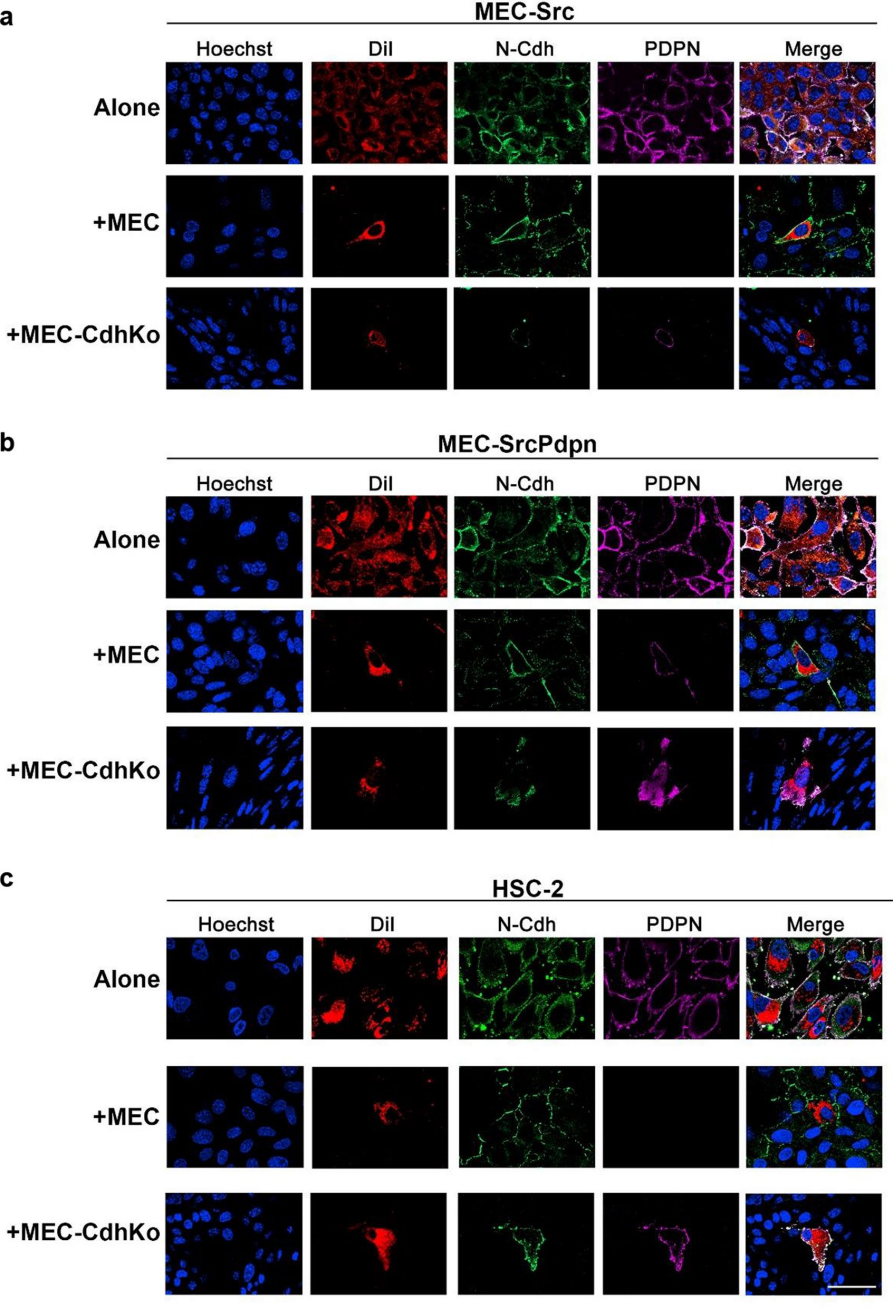
Nontransformed cells inhibit PDPN expression in adjacent transformed cells. Transformed (MEC-Src, HSC-2, MEC-SrcPdpn) cells were stained with DiI (red). These labeled cells were then plated with unlabeled cells of the same type (alone), nontransformed wild type mouse embryonic cells (MEC), or cadherin knockout mouse embryonic cells (MEC-CdhKo) at a 1:300 ratio. Cultures were grown for 24 h before cells were stained with Hoechst (blue), N-Cdh antibody (green), PDPN antibody (purple), and visualized by confocal microscopy as indicated (scale bars = 50 μm)

The PDPN receptor acts as a powerful tumor promoter, and its expression is inhibited by contact normalization [4, 8, 37, 38]. We, therefore, sought to examine the effect of cadherin mediated growth control on PDPN expression. Results from immunofluorescent analysis indicate that nontransformed MECs inhibited PDPN expression as they formed N-Cdh junctions with transformed MEC-Src and HSC-2 cells as shown in Fig. 4a and b.

**Fig. 5:**
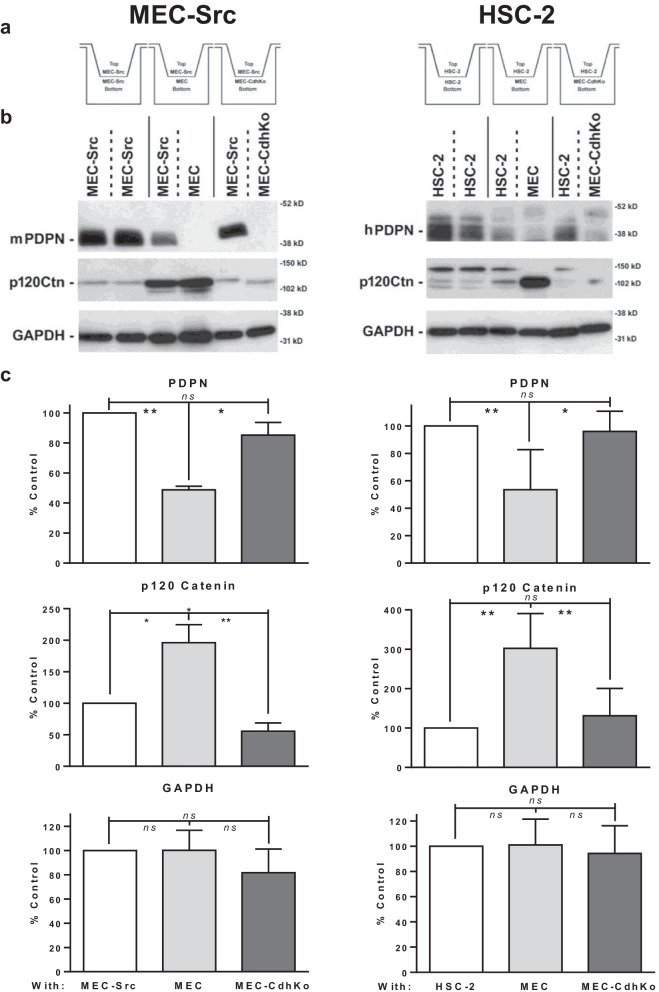
Nontransformed cells decrease PDPN and increase p120-catenin expression in adjacent transformed cells. **a** Src transformed (MEC-Src) or HSC-2 cells, and nontransformed cells with (MEC) or without (MEC-CdhKo) N-Cdh expression were above or beneath a porous membrane at a 1:3 ratio, respectively. This membrane permits junctional communication but prevents actual mixing of the cell populations. **b** Each population of transformed and nontransformed cells in this layered culture system were separately harvested 24 h after plating, and analyzed for PDPN, p120-catenin, and GAPDH expression by Western blot analysis as indicated. **c** Western blot results were quantitated and shown as percent of Src transformed (MEC-Src) cells plated over MEC-Src cells or percent of HSC-2 cells plated over HSC-2 cells (mean + SEM, n = 3, n = 5). Single asterisk, double asterisks, and ns indicate differences with *p* < 0.05, *p* < 0.01, or *p* > 0.05, respectively, by t-test as indicated

A layered culture system was employed to quantitate the effect of contact normalization on PDPN expression. This system allows transformed and nontransformed cells to be maintained as separate populations while forming junctions with each other through porous membranes. This technology allows for transformed and nontransformed cells to be divided into populations that can be quickly separated and analyzed [4, 10, 12]. We utilized this method to quantitate the effect of contact normalization on PDPN expression in MEC-Src cells as shown in Fig. 5a. Western blot analysis from these experiments indicate that wild type MECs decreased PDPN expression in adjacent transformed MEC-Src cells by over 50% as shown in Fig. 5b and c.

Having verified that contact normalization decreased PDPN in Src transformed cells, we utilized the layered culture system to examine the role of N-Cdh in this process. As stated above, wild type MECs inhibited PDPN expression in adjacent Src transformed cells by over 50%. This was significantly more than cadherin deficient MEC-CdhKo cells which inhibited PDPN expression in adjacent MEC-Src cells by less than 20% as shown in Fig. 5b and c. These data indicate that cadherin junctions were required for nontransformed cells to inhibit PDPN expression in neighboring transformed cells.

PDPN expression correlates with increased OSCC metastasis and fatality [39, 40]. We utilized the layered culture system to determine if contact normalization decreased PDPN expression in HSC-2 OSCC cells. Results from Western blot analyses indicated that wild type MECs decreased PDPN expression in adjacent HSC-2 cells by over 30%. In contrast, cadherin deficient MEC-CdhKo cells inhibited PDPN expression in adjacent HSC-2 cells by less than 10% as shown in Fig. 5b and c. Thus, N-Cdh appears to form junctions that mediate contact normalization and decrease PDPN expression in both Src transformed mouse and human OSCC cells.

Having found that heterocellular N-Cdh junctions were required for contact normalization and suppressed PDPN expression in transformed cells, we sought to determine if PDPN expression was sufficient for transformed cells to override growth inhibition by contact normalization. We generated MEC-Src cells with constitutive exogenous PDPN expression for these assays. PDPN, N-Cdh, and active Src kinase expression was verified in these MEC-SrcPdpn cells by Western blot analysis as shown in Fig. 2a. This forced PDPN expression was not reduced by contact with nontransformed MECs in coculture as shown in Fig. 4c. These MEC-SrcPdpn cells grew over 75 fold more in coculture with nontransformed MECs than Src transformed MEC-Src cells without forced PDPN expression as shown in Fig. 1. These data indicate that forced PDPN expression enabled transformed cells to override growth inhibition by N-Cdh mediated contact normalization.

### Contact normalization stabilizes p120-catenin expression in transformed cells

**Fig. 6:**
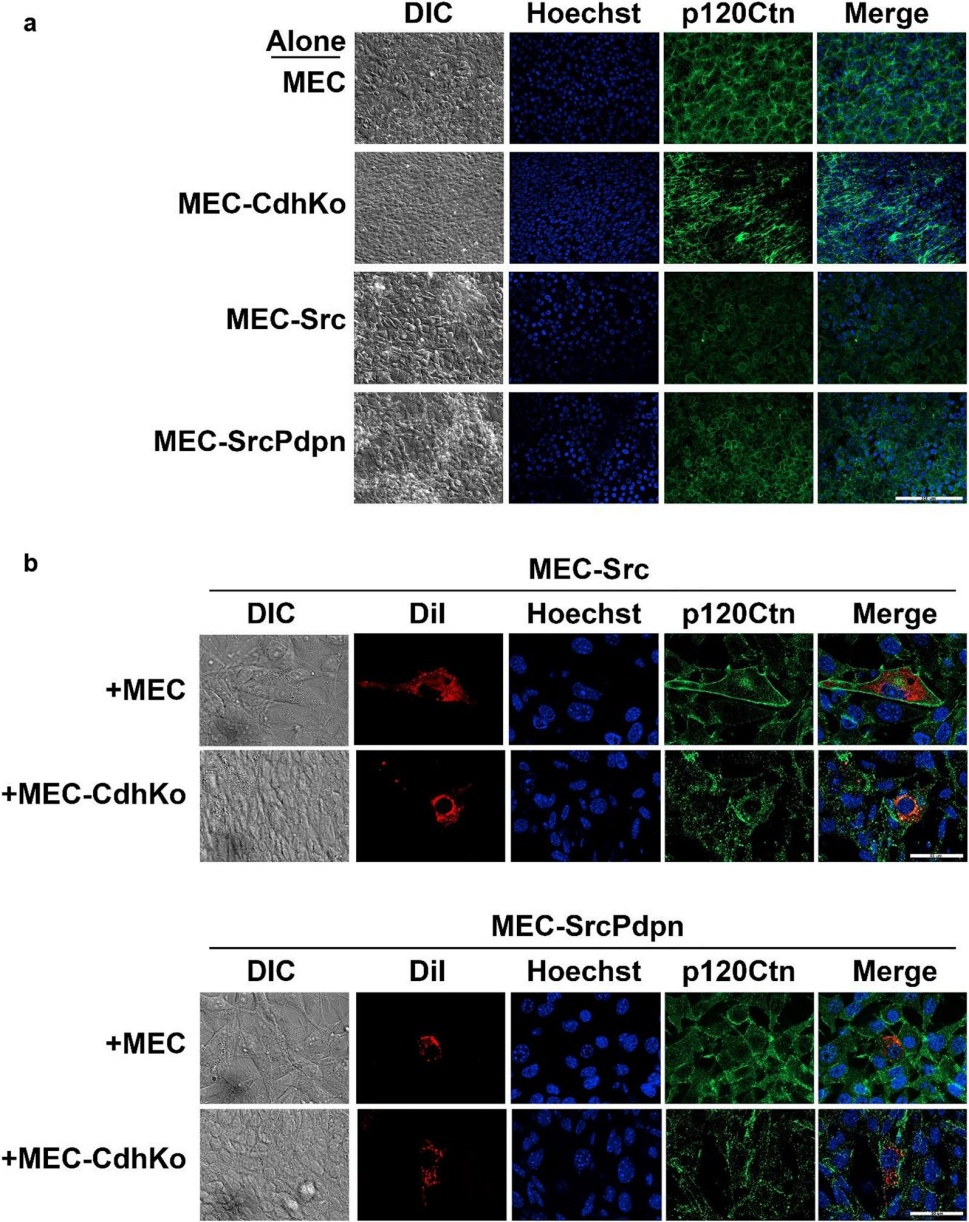
Nontransformed cells stabilize p120-catenin expression in adjacent Src transformed cells. **a** Src transformed (MEC-Src and MEC-SrcPdpn), nontransformed wild type (MEC), and cadherin knockout (MEC-CdhKo) mouse embryonic cells were plated alone (scale bar = 200 microns). **b** Src transformed cells without (MEC-Src) and with (MEC-SrcPdpn) forced PDPN expression stained with DiI (red) were plated with nontransformed wild type (MEC) or cadherin deficient (MEC-CdhKo) cells at a 1:30 ratio. Cultures were grown for 24 h, stained with Hoechst (blue) and p120 catenin antibody (green), and visualized by confocal microscopy as indicated (scale bars = 50 μm)

Cadherins interact with catenins to form functional adherens junctions. In particular, p120-catenin enhances cadherin stability [41, 42]. Accordingly, p120-catenin expression can decrease tumor progression and metastasis of many cancer types including OSCC [43]. We, therefore, examined the effect of contact normalization on p120-catenin expression in this study. Western blot analysis found over 70% less p120-catinin expression in Src transformed (MEC-Src) and cadherin deficient (MEC-CdhKo) cells compared to nontransformed MECs as shown in Fig. 2. This result was confirmed by immunofluorescent analysis as shown in Fig. 6a. These results indicate a strong connection between p120-catenin and adherens junction stability. Interestingly, p120-catenin expression was increased by exogenous PDPN expression in transformed cells, which might be a consequence of PDPN altering the effects of cadherin signaling (see Discussion).

We utilized the layered culture system to quantitate the effect of contact normalization on p120-catenin expression. Western blot analysis from these experiments indicate that MECs increased p120-catenin expression in adjacent transformed MEC-Src cells and HSC-2 cells by about twofold and threefold, respectively, as compared to transformed cells cultured alone as shown in Fig. 5. This result was confirmed by immunofluorescent analysis as shown in Fig. 6. In addition, cadherin deficient MEC-CdhKo cells failed to increase p120-catenin expression in adjacent transformed MEC-Src or HSC-2 cells as shown in Figs. 5 and 6. Taken together, these results indicate that nontransformed cells form cadherin junctions with adjacent transformed cells to stabilize p120-catenin expression and decrease PDPN expression in order to inhibit tumor cell proliferation.

### Contact normalization affects Src-transformed cell transcriptome

**Fig. 7:**
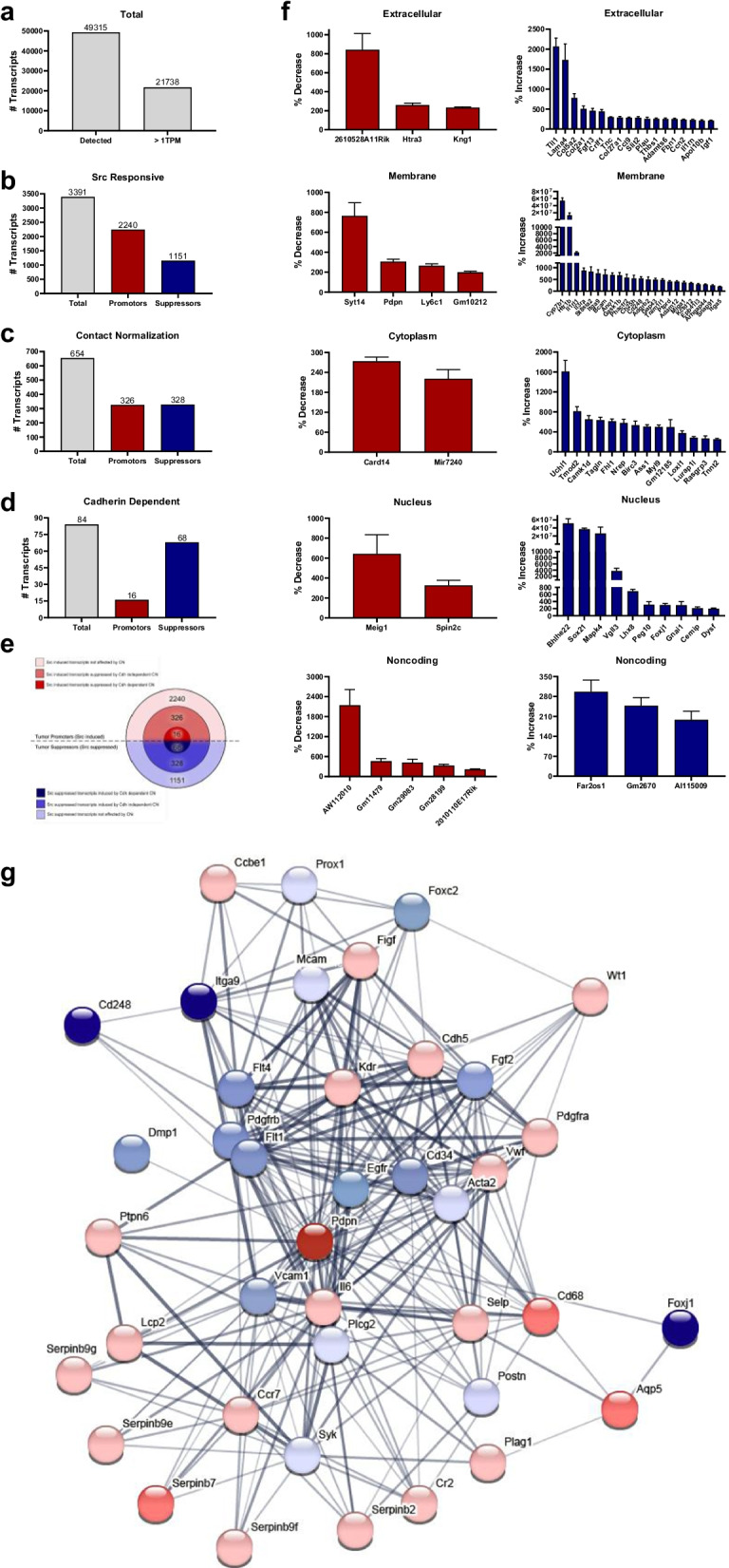
Contact normalization affects Src-transformed cell transcriptome. RNA-seq analysis was performed on Src transformed (MEC-Src) cells cultured with themselves, nontransformed (MEC) cells, and cadherin deficient (MEC-CdhKo) cells in the layered culture system. **a** A total of 49,315 unique gene transcripts were detected, with 21,738 expressed at a level of at least 1 transcript per million (TPM) in any of the cell types. **b** Analysis of transcripts from nontransformed (MEC) and Src transformed (MEC-Src) cells cultured with themselves indicate that oncogenic Src kinase altered the expression of 3391 transcripts by at least threefold (*p* < 0.05 by t-test, n = 3). Src transformation increased the expression of 2240 genes and decreased the expression of 1151 genes as indicated. **c** Transcripts from Src transformed (MEC-Src) cells cultured with themselves were compared with transcripts from MEC-Src cells cultured with nontransformed (MEC cells) to identify genes affected by contact normalization. Six hundred and fifty four out of the 3391 genes affected by Src transformation were inversely affected by at least 40% (*p* < 0.08 by t-test, n = 3). Contact normalization increased the expression of 326 genes that were originally inhibited by Src, and decreased the expression of 328 genes that were originally increased by Src. **d** Transcripts from Src transformed (MEC-Src) cells cultured with nontransformed (MEC) cells were compared with transcripts from MEC-Src cells cultured with cadherin deficient (MEC-CdhKo) to identify genes affected by contact normalization in a cadherin dependent manner. Eighty four of the 326 genes affected by contact normalization were differentially affected by cadherin expression by at least twofold (*p* < 0.05 by t-test, n = 3). Contact normalization decreased the expression of 16 of these genes, and increased the expression of 68 genes. **e** Onion diagram of genes affected by Src transformation and contact normalization. Src increased the expression of 2240 transcripts (light red), and decreased the expression of 1151 transcripts (light green) that were not affected by contact normalization (*p* < 0.05 by t-test, n = 3). The expression of 326 Src induced transcripts were suppressed (medium red) and 328 Src suppressed transcripts were induced by contact (medium blue) with by cadherin deficient nontransformed (MEC-CdhKo) cells (*p* < 0.08 by t-test, n = 3). The expression of 16 Src induced transcripts were suppressed (dark red) and 68 Src suppressed transcripts (dark blue) were induced by contact with cadherin expressing (MEC) nontransformed cells, but not cadherin deficient (MEC-CdhKo) cells (*p* < 0.05 by t-test, n = 3). **f** Genes affected by contact normalization in a cadherin dependent manner were categorized by cellular location. Transcript expression levels are shown as the percent of Src transformed (MEC-Src) cultured with nontransformed cadherin competent (MEC) cells compared with Src transformed cells cultured with nontransformed cadherin deficient (MEC-CdhKo) cells (mean + SEM, n = 3). The expression of putative tumor promoters and suppressors were decreased and increased by contact normalization, respectively. **g** PDPN interacts with 40 gene products affected by Src. STRING protein–protein interaction analysis reveals network nodes representing proteins (circles) and edges (lines) indicating physical and functional protein associations. PDPN interacts with 18 proteins encoded by transcripts increased (light red) and 6 transcripts suppressed (light blue) by Src, but not affected by contact normalization. Cadherin deficient and competent nontransformed cells decreased the expression of 3 Src induced transcripts (medium red), and increased the expression of 8 transcripts (medium blue) suppressed by Src that encode proteins interacting with PDPN in adjacent transformed cells. Cadherin competent cells decreased the expression of PDPN induced by Src (dark red), and increased the expression of 3 transcripts suppressed by Src (dark blue) that were not affected by cadherin deficient cells. Edge line thickness represent interaction strength score

RNA-Seq analysis was employed to investigate how contact normalization affects gene expression in Src transformed cells. Src transformed (MEC-Src) cells were cultured with themselves, nontransformed (MEC) cells, or cadherin deficient (MEC-CdhKo) cells in the layered culture system. A total of 49,315 unique gene transcripts were detected, with 21,738 expressed at a level of at least 1 transcript per million (TPM) in any of the cell types as shown in Fig. 7a. Comparison of transcripts from nontransformed (MEC) and Src transformed (MEC-Src) cells cultured with themselves indicate that oncogenic Src kinase altered the expression of 3391 transcripts by at least threefold as shown in Fig. 7b and e. Src increased the expression of 2240 of these genes and decreased the expression of 1151. Transcripts from Src transformed (MEC-Src) cells cultured with themselves were compared with transcripts from MEC-Src cells cultured with nontransformed (MEC cells) to identify genes affected by contact normalization. This comparison found that 654 out of the 3391 genes affected by Src transformation were inversely affected by at least 40%. Contact normalization increased the expression of 326 genes that were originally inhibited by Src, and decreased the expression of 328 genes that were originally increased by Src genes as shown in Fig. 7c and e. Transcripts from Src transformed (MEC-Src) cells cultured with nontransformed (MEC) cells were compared with transcripts from MEC-Src cells cultured with cadherin deficient (MEC-CdhKo) cells to identify genes affected by contact normalization in a cadherin dependent manner. This comparison found that 84 of the 326 genes affected by contact normalization were differentially affected by cadherin expression by at least twofold. Cadherin dependent contact normalization decreased the expression of 16 of these genes, and increased the expression of 68 genes as shown in Fig. 7d and e.

Gene ontology accession terms were used to categorize cellular location of transcripts affected by contact normalization in a cadherin dependent manner as shown in Fig. 7f. Of the 16 transcripts increased by Src and decreased by cadherin dependent contact normalization, 3 code for extracellular proteins, 4 localize to the plasma membrane, 2 are cytoplasmic, 2 localize to the nucleus, and 5 are noncoding RNAs. Of the 68 transcripts decreased by Src and increased by cadherin dependent contact normalization, 18 code for extracellular proteins, 23 localize to the plasma membrane, 14 are cytoplasmic, 10 localize to the nucleus, and 4 are noncoding RNAs. Gene products affected by Src and contact normalization that interact with PDPN were identified by STRING protein interaction analysis. PDPN interacts physically or functionally with 40 of these gene products as shown in Fig. 7g. Src kinase activity increased and decreased the expression of transcripts encoding 22 and 18 of these proteins, respectively. For example, expression of transcripts encoding the tumor promoters Serpinb7 [44], Aqp5 [45, 46], and Cd68 [47] was induced by Src and suppressed by contact normalization. Conversely, expression of transcripts encoding the tumor suppressors Dmp1 [48] and Foxj1 [49, 50] was suppressed by Src and increased by contact normalization. In addition, PDPN was found to interact functionally with the tumor promoters Kdr/Vegfr2 [51] and Wt1 [52] which were induced by Src, but not affected by contact normalization.

## Discussion

Contact normalization is a widespread and powerful process. Nontransformed cells can normalize the growth of adjacent cells transformed by a variety of chemical agents and oncogenes including Ras, Myc, and Src. Transformed cells survive contact normalization; they assume a normal morphology and phenotype, but retain their tumorigenic potential which can be activated after communication with nontransformed cells is interrupted. This process has been attributed to dormant cancer cells that are suppressed for years, or even decades, before they emerge to cause malignancies in many tissues including skin, breast, and intestine. Transformed cells must override contact normalization before they can realize their malignant and metastatic potential [6–9, 53].

In this study, we utilized well characterized connexin knockout cells to identify junctions required for contact normalization independent of gap junctional communication [4, 5, 10–12]. Nontransformed cells with endogenous N-Cdh expression effectively suppressed the growth of cocultured Src transformed MECs and human OSCC cells in this system. We also show that heterocellular N-Cdh junctions formed between these nontransformed and transformed cells. Moreover, nontransformed cells could no longer suppress neighboring transformed cell growth after N-Cdh expression in nontransformed cells was ablated by CRISPR. These data indicate that N-Cdh mediated contact normalization in these cells.

Cadherins exert complex effects on tumor progression. E-Cdh acts as a tumor suppressor in epithelial cells. “Cadherin switching” from E-Cdh to N-Cdh expression is involved in EMT (epithelial to mesenchymal transition) associated with tumor progression. N-Cdh is often expressed by tumor stromal cells including CAFs which can enhance or inhibit tumor expansion [54–56]. Like E-Cdh, N-Cdh can also act as a tumor suppressor. Both E-Cdh and N-Cdh utilize catenins to normalize the actin cytoskeleton and cell behavior [15–17, 23, 24]. In this study, p120-catenin destruction accompanied oncogenic kinase activity, and this effect was ameliorated by contact with nontransformed cells. We employed a layered culture system to find that contact normalization doubled p120-catenin levels in transformed cells. This effect required cadherin expression since nontransformed N-Cdh knockout cells did not augment p120-catenin expression in cocultured transformed cells. However, forced PDPN expression also increased p120-catenin expression in transformed cells. This effect might arise from a nexus of common function; for example, both PDPN and N-Cdh can promote individual and collective tumor cell motility [15, 16, 37, 38, 57]. Future studies would be well to investigate the effect of heterocellular cadherin junctions between transformed and nontransformed cells on catenin stability and downstream signaling events.

**Fig. 8:**
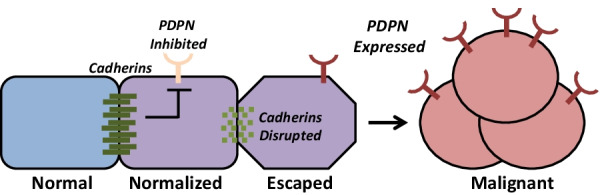
Nontransformed cells form heterocellular cadherin junctions with adjacent transformed cells to inhibit PDPN expression and cancer progression. Cadherins are required for this process of contact normalization, and PDPN expression is sufficient to override this form of growth control

Contact normalization is a dynamic process that has survived species divergence, as evidenced by nontransformed mouse cells controlling the growth of neighboring human OSCC cells in this study. Taken together, results from this study indicate that nontransformed cells utilize N-Cdh to inhibit PDPN expression in order to suppress the growth of adjacent transformed cells as shown in Fig. 7. Src phosphorylates the p130Cas/BCAR1 adaptor protein to induce PDPN expression in order to promote tumor cell expansion, and contact inhibition reverses this process [4, 37]. Quantitation from the layered culture system used in this study indicate that contact normalized cells express about half of the PDPN levels seen in unopposed transformed cells. Moreover, this effect relies on cadherin expression since PDPN expression is not suppressed in transformed cells cultured with nontransformed cadherin deficient cells. Most importantly, data from this study indicate that PDPN signaling is sufficient to override contact normalization since the growth of transformed cells with forced PDPN expression was not inhibited by nontransformed cells, regardless of cadherin expressionas shown in Fig. 8.

We utilized RNA-Seq in this study to identify genes affected by Src transformation and contact normalization. Src transformation affected approximately 16% of the transcriptome, increasing the expression of about 66% of these transcripts. This is consistent with results from studies by microarray analysis which indicate that between 8% [12] to 25% [4] of the MEC transcriptome was affected by Src transformation, and that Src increased about 66% of these transcripts. However, while these microarray analyses found that 0.01–0.02% of the transcriptome in Src transformed MECs was affected by contact normalization [4, 12], RNA-Seq analysis performed in this study found that contact normalization altered the expression of about 3% of the transcriptome in these cells. This increase might be attributed to the improved sensitivity that RNA-Seq analysis provides, including enhanced detection of different transcript isoforms, over microarray assays [58]. We utilized this approach to find that cadherin dependent contact normalization alters the expression of about 0.4% of the transcriptome in Src transformed cells. Therefore, only about 10% of the genes affected by contact with nontransformed cells rely on cadherin mediated communication. Heterotypic cadherin junctions increased or decreased the expression of about 80% and 20% of these transcripts, respectively. Most notably, Src inhibited expression of the Fhl1 tumor suppressor and induced expression the Pdpn tumor promoter, and this effect was reversed by contact normalization as previously reported [4, 11, 12]. However, we report here that this effect of contact normalization relies on heterocellular cadherin junctions between transformed and nontransformed cells. Interestingly, previous studies and STRING analysis indicate that PDPN expression was coordinated with the expression of other tumor promoters, specifically Kdr/Vegfr2 [4, 51, 59] and Wt1 [52], that were induced by oncogenic Src kinase activity. This scenario suggests that functional relationships between these oncoproteins contribute to cancer progression as transformed cells escape contact normalization.

## Conclusions

Results from this study indicate that cadherins can mediate contact normalization. Nontransformed cells form cadherin junctions with neighboring transformed cells to inhibit tumor cell growth, and PDPN can override this form of growth control. PDPN is a transmembrane mucin receptor that has been identified as a tumor promotor expressed by a variety of cancers and associated CAFs [38, 40, 60]. For example, PDPN has emerged as a functionally relevant biomarker and potential chemotherapeutic target to prevent and treat oral cancer. PDPN expression is elevated in the majority of OSCC cells, and is a strong indicator of tumor aggression. PDPN is also induced early in OSCC progression and can be used to identify premalignant lesions that are bound to develop into malignancies [39, 40, 61]. In addition to OSCC, PDPN promotes a variety of other cancers including mammary carcinoma [62, 63], glioma [64–67], melanoma [59, 68, 69], ovarian cancer [70], and pulmonary adenocarcinoma [71–73]. Indeed, PDPN has been identified as an enticing target for chemotherapy [74, 75]. The roles of cadherins and PDPN in contact normalization described in this study should yield insight into fundamental mechanisms that affect the general process of cancer development and progression at the cellular level of the tumor microenvironment.

## Data Availability

The datasets used and/or analyzed during the current study are available from the corresponding author on reasonable request. RNA-Seq data from this study can be accessed at Sequence Read Archive (SRA) submission: SUB10367693.

## Abbreviations

CAFs: Cancer associated fibroblasts
N-Cdh: N-cadherin
OSCC: Oral squamous cell carcinoma
